# Elongin C (*ELOC*/*TCEB1*)-associated von Hippel–Lindau disease

**DOI:** 10.1093/hmg/ddac066

**Published:** 2022-03-21

**Authors:** Avgi Andreou, Bryndis Yngvadottir, Laia Bassaganyas, Graeme Clark, Ezequiel Martin, James Whitworth, Alex J Cornish, Genomics England Research Consortium, Richard S Houlston, Philip Rich, Catherine Egan, Shirley V Hodgson, Anne Y Warren, Katie Snape, Eamonn R Maher

**Affiliations:** Department of Medical Genetics, University of Cambridge, Cambridge Biomedical Campus, Cambridge CB2 0QQ, UK; Department of Medical Genetics, University of Cambridge, Cambridge Biomedical Campus, Cambridge CB2 0QQ, UK; Department of Medical Genetics, University of Cambridge, Cambridge Biomedical Campus, Cambridge CB2 0QQ, UK; Department of Medical Genetics, University of Cambridge, Cambridge Biomedical Campus, Cambridge CB2 0QQ, UK; Stratified Medicine Core Laboratory NGS Hub, Cambridge Biomedical Campus, Cambridge CB2 0QQ, UK; Department of Medical Genetics, University of Cambridge, Cambridge Biomedical Campus, Cambridge CB2 0QQ, UK; Stratified Medicine Core Laboratory NGS Hub, Cambridge Biomedical Campus, Cambridge CB2 0QQ, UK; Department of Medical Genetics, University of Cambridge, Cambridge Biomedical Campus, Cambridge CB2 0QQ, UK; Division of Genetics and Epidemiology, The Institute of Cancer Research, Sutton, Surrey SM2 5NG, UK; Division of Genetics and Epidemiology, The Institute of Cancer Research, Sutton, Surrey SM2 5NG, UK; Department of Neuroradiology, St. George’s University Hospitals NHS Foundation Trust, London SW17 0QT, UK; NIHR Biomedical Research Center at Moorfields Eye Hospital NHS Foundation Trust and UCL Institute of Ophthalmology, London, UK; South West Thames Regional Genetics Service, St George's University Hospitals NHS Foundation Trust, London, UK; Department of Histopathology, Cambridge University NHS Foundation Trust, Cambridge CB2 OQQ, UK; South West Thames Regional Genetics Service, St George's University Hospitals NHS Foundation Trust, London, UK; St George's University of London, UK; Department of Medical Genetics, University of Cambridge, Cambridge Biomedical Campus, Cambridge CB2 0QQ, UK

## Abstract

Around 95% of patients with clinical features that meet the diagnostic criteria for von Hippel–Lindau disease (VHL) have a detectable inactivating germline variant in *VHL*. The VHL protein (pVHL) functions as part of the E3 ubiquitin ligase complex comprising pVHL, elongin C, elongin B, cullin 2 and ring box 1 (VCB-CR complex), which plays a key role in oxygen sensing and degradation of hypoxia-inducible factors. To date, only variants in *VHL* have been shown to cause VHL disease. We undertook trio analysis by whole-exome sequencing in a proband with VHL disease but without a detectable *VHL* mutation. Molecular studies were also performed on paired DNA extracted from the proband’s kidney tumour and blood and bioinformatics analysis of sporadic renal cell carcinoma (RCC) dataset was undertaken. A *de novo* pathogenic variant in *ELOC* NM_005648.4(ELOC):c.236A>G (p.Tyr79Cys) gene was identified in the proband. *ELOC* encodes elongin C, a key component [C] of the VCB-CR complex. The p.Tyr79Cys substitution is a mutational hotspot in sporadic VHL-competent RCC and has previously been shown to mimic the effects of pVHL deficiency on hypoxic signalling. Analysis of an RCC from the proband showed similar findings to that in somatically *ELOC*-mutated RCC (expression of hypoxia-responsive proteins, no somatic *VHL* variants and chromosome 8 loss). These findings are consistent with pathogenic *ELOC* variants being a novel cause for VHL disease and suggest that genetic testing for *ELOC* variants should be performed in individuals with suspected VHL disease with no detectable *VHL* variant.

## Introduction

Genetic studies of rare familial cancer syndromes have provided important insights into cancer biology and mechanisms of human disease. This is exemplified by von Hippel–Lindau disease/syndrome (VHL) (MIM:193300), an autosomal dominant multisystem cancer predisposition disorder characterized by predisposition to retinal and central nervous system haemangioblastomas, clear cell renal cell carcinoma (ccRCC), phaeochromocytoma/paraganglioma (PPGL), non-secretory pancreatic neuroendocrine tumours and endolymphatic sac tumours (1,2). The cardinal features for a diagnosis of VHL disease were defined in the early 1960s: two or more retinal or central nervous system haemangioblastomas or a haemangioblastoma and ccRCC or phaeochromocytoma or a positive family history of VHL disease and a single tumour (haemangioblastoma, ccRCC or phaeochromocytoma) (3).

The incidence of VHL disease is ~1 in 36 000 live births (4) and following clinical descriptions of large affected families and genetic linkage studies mapped a gene to chromosome 3p25-26 with no evidence of locus heterogeneity (5). The von Hippel–Lindau tumour suppressor gene (TSG) [*VHL* (MIM: 608537)] was identified in 1993 (6) and over 1000 pathogenic germline and somatic *VHL* variants have now been described (7). Around 95% of individuals with clinical features that meet the diagnostic criteria for VHL disease have an inactivating germline *VHL* variant detectable by standard molecular genetic testing. Recently some ‘*VHL* mutation-negative’ cases have been demonstrated to have mosaicism, promoter region variants or an intronic *VHL* mutation, but no other genes have been reported to cause VHL disease (8,9). Germline *VHL* pathogenic variants may also be detected in individuals with a clinical diagnosis of VHL disease (e.g. apparently sporadic haemangioblastoma or with familial PPGL), and rare biallelic missense variants have been shown to cause autosomal recessive polycythaemia (10,11).

Tumours from individuals with VHL disease show somatic inactivation of the wild-type allele consistent with the Knudsen two-hit model of tumourigenesis (12). Furthermore, in sporadic ccRCC and haemangioblastomas, somatic biallelic inactivation of the *VHL* TSG occurs as a critical and early event in tumourigenesis (13,14). The identification of the *VHL* TSG led to the discovery of its role in the pathogenesis of sporadic ccRCC and the fundamental role of the gene product in cellular oxygen sensing (1,15). Tumours with *VHL* TSG inactivation are highly vascular and demonstrate hypoxia-independent activation of the hypoxic gene response pathway targets, with overexpression of angiogenic (e.g. vascular endothelial growth factor and platelet-derived growth factor beta polypeptide) and oncogenic (cyclin D1) factors (16,17). The VHL protein (pVHL) has a critical role in regulating the expression of the α-subunits of the hypoxia-inducible transcription factors, HIF-1 and HIF-2, that regulate the cellular response to hypoxia such that pVHL functions as the target-binding component of an E3 ubiquitin ligase complex comprising pVHL, elongin C, elongin B, cullin 2 (CUL2) and ring box 1 (RBX1), abbreviated as the VCB-CR complex (15,18,19). To date, germline mutations in non-VHL components of the VCB-CR complex have not been reported. Herein, we describe the association of VHL disease-like phenotype with a pathogenic variant in the *ELOC* gene encoding the elongin C protein, which binds to pVHL.

## Results

### Case report

**Figure 1:**
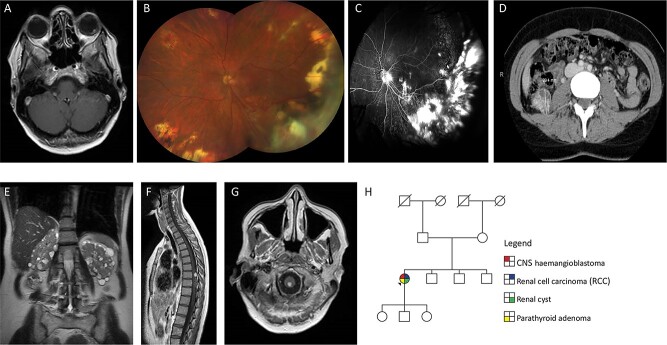
(**A**) Axial T1-weighted post-contrast image through the orbits shows a retinal angioma in the left globe. (**B**) Colour fundus photograph of left eye at most recent clinic visit showing areas of previous laser and cryotherapy treatment with dragging of optic nerve vessels towards inferotemporal quadrant. Haemangiomas present in macula and nasal quadrant. Multiple peripheral chorioretinal scars related to previously treated haemangiomas. Superotemporal vessels with perivascular exudate. Right eye normal (not shown). Visual acuity Right 6/5 and Left 6/12. (**C**) Fluorescein angiogram performed 4 years previously showing areas of scarring and retinal detachment related to exudation and effect of treatment. Optic nerve leak related to the effect of traction and glial proliferation with areas of hyperfluorescence in the macula related to new small haemangiomas, visible anterior to the internal limiting membrane on OCT scans (not shown). Right eye normal (not shown). (**D**) CT scan showing 29 mm diameter RCC in right kidney. (**E**) Coronal T2-weighted image through the abdomen shows numerous small cysts in both kidneys. (**F**) Sagittal T1-weighted post-contrast image of the spinal cord shows a solid enhancing haemangioblastoma with associated hypertrophied vessels on the dorsal surface of the spinal cord. (**G**) Axial T1-weighted post-contrast image through the cervicomedullary junction shows a small solid haemangioblastoma. (**H**) Family pedigree of sporadic case of VHL.

A 37-year-old female of Northern European origin presented with two left retinal haemangioblastomas that were treated by laser treatment (Fig. 1A–C). Two years later, she developed an RCC and cyst of the right kidney which were treated by partial right nephrectomy (Fig. 1D and E). At the age of 47 years, a further RCC was detected in the left kidney and was treated by cryoablation. A spinal haemangioblastoma was removed at the age of 52 years (Fig. 1F) and a haemangioblastoma at the cervicomedullary junction remains under surveillance (Fig. 1G). Before developing features indicative of VHL disease, she had presented with Henoch-Schonlein purpura at the age of 23 years and underwent unilateral parathyroidectomy for two parathyroid adenomas at the age of 28 years. Family history was unremarkable with both parents, three siblings and three children not reporting any features of the VHL disease (Fig. 1H).

**Figure 2:**
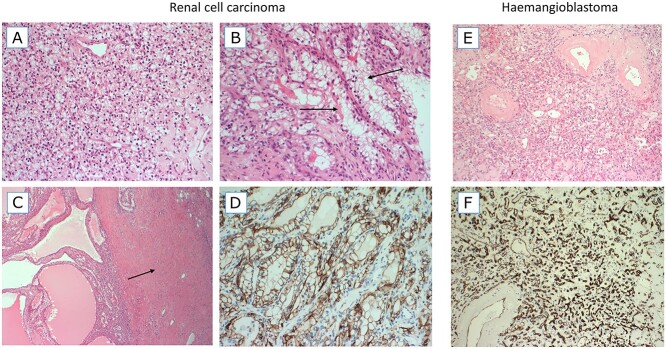
Haematoxylin and eosin (H&E)-stained images (**A**–**C**) and CA-IX staining image from the RCC. H&E and CD34-stained images from the haemangioblastoma. (A) An area with typical features of a ccRCC, composed of a sheet of small cells with clear cytoplasm and a delicate background vascular network. (B) Focus on branching tubules in which the tumour cells have more voluminous clear cytoplasm (arrows). (C) A cystic area of the RCC tumour (left) and a dense band of leiomyomatous (muscular) stroma (right, arrow). (**D**) The tumour was diffusely positive for CA-IX, a classic marker of HIF up-regulation. (**E**) The haemangioblastoma tumour is composed of very small cells with clear cytoplasm and a background vascular network. Larger blood vessels have thickened hyalinized walls. (**F**) The vascular network of haemangioblastoma is highlighted by CD34 immunohistochemistry.

Pathological examination of the right partial nephrectomy sample was consistent with ccRCC, staged as Fuhrman grade 2 (g2) pT1 NX. Sections from the right renal cyst showed fibrous walled cysts lined by regular clear epithelial cells with small nuclei (Fig. 2A–C). The tumour was diffusely positive for cytokeratin AE1/3 (AE1/3), carbonic anhydrase 9 (CA-IX) (Fig. 2D) and Vimentin and showed focal positivity for cytokeratin 7 (CK7) and RCC (weak) on immunohistochemistry. Fumarate hydratase staining was retained and 2-succinocysteine staining was negative. The appearances were considered typical of those seen in VHL. In addition, the presence of a leiomyomatous stroma and occasional branched tubular structures lined by cells with voluminous cytoplasm, features of RCC with somatic *ELOC* variants, were noted focally in the RCC (Fig. 2B) (20,21). The spinal tumour showed features of a haemangioblastoma and was positive for inhibin, Vimentin, S100 protein and CA-IX expression but was negative for AE1/3, paired box gene 8 protein and CK7. Cluster of differentiation 31 protein and CD34 stains highlighted a network of vascular structures (Fig. 2E and F).

**Figure 3:**
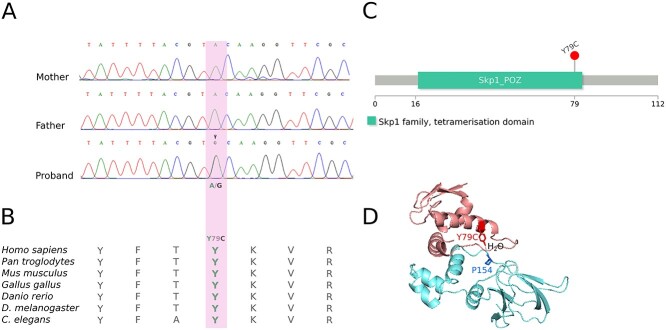
(**A**) Direct (Sanger) sequencing in trio shows the presence of the *ELOC* c.236A>G (p.Tyr79Cys) variant in the proband and absence from the parents. (**B**) Evolutionary conservation. Tyrosine at codon 79 (Y79) is evolutionary conserved across vertebrates and invertebrates (22). (**C**) ELOC domains. The Y79C variant (p.Tyr79Cys) is in the tetramerization domain of the *ELOC* gene (23). (**D**) ELOC Y79C-VHL interaction. Tyr79 mediates a hydrogen bond with Pro154 of VHL via a water molecule; adapted from (25). The X-ray crystallographic structure of the ELOC/VHL complex was downloaded from the Protein Data Bank (PDB:4WQO) (26). Molecules other than ELOC and VHL were removed from the structure for clarity.

Routine diagnostic testing by Sanger sequencing and multiplex ligation-dependent probe amplification (MLPA) for a germline *VHL* variant showed no abnormality, and after informed written consent, the proband and her parents underwent research testing. Whole-exome sequencing (WES) and whole-genome sequencing (WGS) were performed. No candidate *VHL* variants were detected in the proband, but trio analysis identified 16 rare variants (gnomAD maximum allele frequency ≤ 0.5%) (Supplementary Material, Table S1) that were not detectable in either parent. A *de novo* missense variant in *ELOC* NM_005648.4(ELOC):c.236A>G (p.Tyr79Cys) was identified. Direct (Sanger) sequencing validated the presence of the *de novo ELOC* variant in the proband (Fig. 3A). Tyrosine at codon 79 (Y79) is evolutionary conserved across vertebrates and invertebrates (Fig. 3B) (22) and is located in the tetramerization domain of the *ELOC* gene (Fig. 3C) (23). Elongin C Tyr79 residue is known to form a critical hydrogen bond with the Pro154 residue within the pVHL alpha domain (24–26) (Fig. 3D). NM_005648.4(ELOC):c.236A>G(p.Tyr79Cys) was not seen in 76 156 genomes catalogued by gnomAD (v3.1). Deep intronic and promoter variants, described previously in VHL disease or erythrocytosis, were excluded from the proband (Supplementary Material, Table S2).

**Figure 4:**
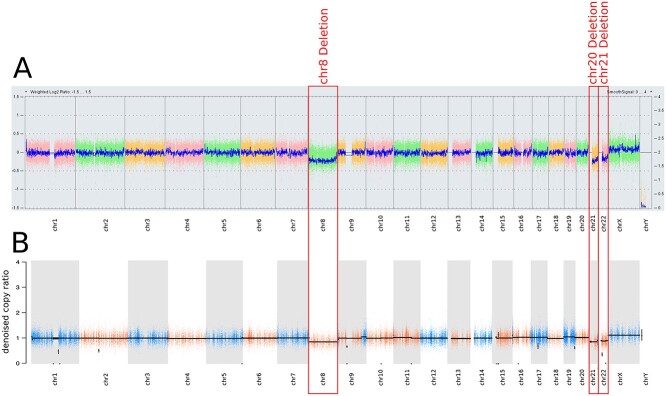
(**A**) aCGH (750k array*)* of germline/tumour pair showed monosomy for chromosomes 8, 21, 22 (in ~20% of cells). (**B**) Targeted WES of germline/tumour pair identified in terms of copy number profile. This shows broad losses involving the full chromosome 8 and the long arms of chromosomes 21 and 22 (in ~40% of cells, tumour fraction ~47.8%). Any of the known recurrent RCC-related copy number aberrations (i.e. 3p, 9p or 14p losses and 5q or chr7 amplification) were not found.

Microarray-based comparative genomic hybridization (aCGH) performed on the DNA pair extracted from the proband’s right RCC and blood showed evidence of monosomy for chromosomes 8, 21 and 22 and no somatic alterations were commonly seen in ccRCC (i.e. deletion of 3p, 9p, 14q or 5q gain) (27) (Fig. 4A). Paired WES for tumour/blood DNA was analyzed for copy number variants (CNVs) and single-nucleotide variants (SNVs)/indels and was consistent with loss of chromosomes 8, 21 and 22, and no evidence of a somatic *VHL* mutation was found (Fig. 4B). The c.236A>G *ELOC* variant was present in 35% and 46% (46/130 and 33/72) of reads in blood and tumour DNAs, respectively. The allele counts for variant (alternate) and wild-type (reference) alleles in blood being biased towards the wild-type is suggesting mosaicism for the NM-005648.4:c.236A>G (p.Tyr79Cys) variant (Supplementary Material, Fig. S1).

In view of the parathyroid adenomas diagnosed in the proband at an early age, we analyzed for the presence of any variants in genes predisposing to any endocrine neoplasia syndromes. No pathogenic/likely pathogenic SNVs, CNVs or structural variants (SVs) were found in *AIP*, *CDC73*, *CDKN1B*, *MEN1* and *RET*.

### *ELOC* c.236A>G (p.Tyr79Cys) variation in human disease

**Figure 5:**
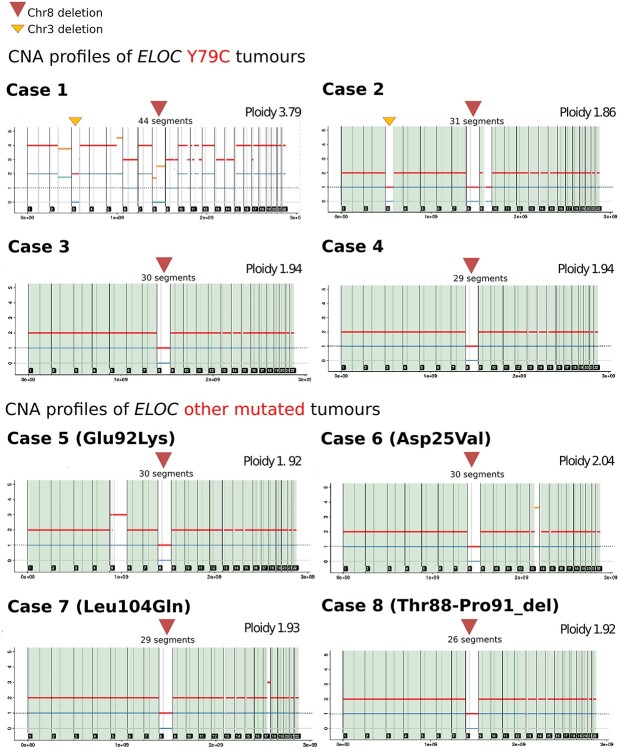
Copy number analysis profiles for the eight RCC tumours with somatic *ELOC* variants from the 100,000 Genomes Project using Battenberg caller (subclonal copy number caller) (56). The *ELOC c.236A>G* (p.Tyr79Cys) missense variant was identified in cases 1–4, cases 5–7 had a non-codon 79 missense *ELOC* variant [NM_005648.4:c.274G>A (p.Glu92Lys), NM_005648.4:c.311T>A (p.Leu104Gln), NM_005648.4:c.74A>T (p.Asp25Val)] and case 8 harboured anin-frame deletion [NM_005648.4:c.261_272del (p.Thr88_Pro91del)].

NM_005648.4(ELOC):c.236A>G (p.Tyr79Cys) was originally described as a somatic variant in six RCCs without VHL inactivation (24), in three cases within The Cancer Genome Atlas (28) and subsequently in five cases from the Memorial Sloan Kettering Cancer Centre cohort (details of specific amino acid substitution at residue 79 were not available) (21) (Supplementary Material, Table S4). To further explore the role of the *ELOC* variants in *VHL-*independent renal tumourigenesis, we searched for additional examples of germline and somatic *ELOC* variants. Germline *ELOC* variants were sought (by Sanger sequencing or WES) in 91 individuals who were recruited in-house with either a VHL-like phenotype *(n* = 91), which was defined as multiple VHL-related tumours, or a single VHL-related tumour plus a family history of a VHL-related tumour (Supplementary Material, Table S3). None of the 91 individuals had evidence of the NM_005648.4(ELOC):c.236A>G (p.Tyr79Cys) variant, or other *ELOC* pathogenic variants. The NM_005648.4(ELOC):c.236A>G (p.Tyr79Cys) variant was also absent from the germline of 78 195 participants in the 100,000 Genomes Project (29), including 1336 individuals with RCC. To further investigate the role of somatic *ELOC* mutations, and in particular c.236A>G in *VHL*-independent renal tumourigenesis, we interrogated the 100,000 Genomes Project dataset (29) and identified 8 of 1336 RCC with a candidate pathogenic *ELOC* somatic variant. Four of the eight RCC tumours had a somatic NM_005648.4(*ELOC*):c.236A>G (p.Tyr79Cys) variant (cases 1–4). Three of the eight RCC had a non-codon 79 missense *ELOC* variant [case 5: NM_005648.4:c.274G>A (p.Glu92Lys), case 6: NM_005648.4:c.74A>T (p.Asp25Val), case 7: NM_005648.4:c.311T>A (p.Leu104Gln)] and the remaining RCC (case 8) harboured a somatic *ELOC* in-frame deletion [NM_005648.4:c.261_272del (p.Thr88_Pro91del)] (Supplementary Material, Table S5). All eight of the RCCs with a candidate somatic pathogenic *ELOC* variant demonstrated chromosome 8 loss and were *VHL* mutation-negative (Fig. 5 and Supplementary Material, Table S5).

## Discussion

We report a germline *de novo* missense substitution NM_005648.4(ELOC):c.236A>G (p.Tyr79Cys) in *ELOC*, previously known as *TCEB1*, in a female who satisfied clinical diagnostic criteria for VHL disease but who did not have a detectable *VHL* mutation. In particular, there was no evidence for intronic or promoter region VHL mutation (Supplementary Material, Table S2) and no evidence for a mosaic *VHL* mutation after analysis of blood and tumour DNA. To our knowledge, the NM_005648.4(ELOC):c.236A>G (p.Tyr79Cys) missense substitution has not been detected as a germline variant previously (20,21,24,28). To date, 20 somatic *ELOC*-mutated RCCs have been reported (21,28) (Supplementary Material, Table S4). Our analysis has not only confirmed the finding of recurrent somatic p.Tyr79Cys substitutions as a hotspot mutational event in sporadic RCC but also has identified additional candidate pathogenic *ELOC* somatic variants that were mostly also missense substitutions. Consistent with DiNatale (21), we found evidence of chromosome 8 deletions in the *ELOC*-mutated sporadic RCCs and also in the RCC associated with a germline *ELOC* mutation.

pVHL has two critical functional domains. Under normoxic conditions, the β-domain binds to two conserved proline residues within the oxygen-dependent degradation domains of the α-subunits of the HIF-1 and HIF-2 transcription factors and targets them for ubiquitin-mediated proteolysis (15,18,19). pVHL deficiency or hypoxia results in HIF-1 and HIF-2 being stably expressed and activating hypoxic-gene response pathways (15–17). The second critical pVHL domain, the α-domain (residues 155–192) (18), interacts with other components of the VCB-CR complex by binding directly to elongin C (18). Germline or somatic *VHL* mutations that disrupt pVHL binding to elongin C result in HIF stabilization and activation of hypoxic-gene response pathways. Within the pVHL α-domain, the Pro154 residue forms a critical hydrogen bond with the elongin C Tyr79 residue (24) (Fig. 3D). Previously, experiments in human embryonic kidney 293 cells have shown that while ELOC-wild-type co-precipitates with pVHL and CUL2, this is greatly reduced for mutant ELOC-Tyr79 (24). Furthermore, ELOC-Tyr79Cys leads to the accumulation of HIF-1α and HIF-2α when compared to tumours without *ELOC* or *VHL* mutations (24). These studies are compatible with our observation of a VHL disease phenotype in an individual with a germline *ELOC* p.Tyr79Cys variant. The previously reported *in vitro* studies are consistent with p.Tyr79Cys functioning as a loss of function variant, and we and others have found that chromosome 8 loss is a feature of *ELOC-*mutated RCC (21). We confirmed this finding in p.Tyr79Cys-mutated RCC and also identified other candidate somatic *ELOC* mutations in sporadic RCC which were also associated with chromosome 8 loss. It is clear that *ELOC* p.Tyr79Cys is a mutation hotspot, but the explanation for this is currently unclear. One possibility is that *ELOC* p.Tyr79 substitutions might disrupt pVHL-related functions of the VBC-CR complex while leaving other functions (e.g. RNA polymerase II elongation) intact and/or there is a requirement for a specific level of ELOC function to promote tumourigenesis, which is similar to the ‘just-right’ signalling model proposed for the APC tumour suppressor function (30).

Though inactivation of the *VHL* and *ELOC* TSGs will both result in dysregulation of hypoxic gene response pathways and other HIF-independent pVHL functions, there will be differences in the effects on other cellular pathways, and this might result in additional or varied presentation of clinical features within patients with a germline *ELOC* mutation. For example, elongin C is known to link SOCS proteins, which are negative feedback inhibitors of cytokine and growth factor-induced signal transduction, to the proteasome and target them for degradation (31). SOCS1 was shown to interact with elongin B, elongin C and Cul2 and to target JAK2, Vav, IRS1 and IRS2 for ubiquitylation and proteasomal degradation (32–34). SOCS2 forms a complex with elongin B and elongin C (SOCS2–elongin C–elongin B complex), which acts as an E3 ubiquitin ligase similar to the VCB-CR complex showing a shared mechanism of ubiquitination between these cullin-dependent E3 ligases (35). Phosphopeptide substrates derived from the growth hormone receptor and the erythropoietin receptor are recognized targets of SOCS2 (31,35,36). Therefore, the reason for specific mutation hotspot (and absence of truncating mutations) in *ELOC* might relate to the fact that these alterations affect the interaction with VHL but not with other proteins such as SOCS1/2 (or other pVHL-unrelated ELOC functions). The overlapping but distinct functional effects of pVHL and ELOC inactivation appear to be reflected in differing patterns of somatic copy number events and mutations in *VHL*- and *ELOC*-mutated RCC. RCCs with germline and somatic *VHL* TSG mutations have a high frequency of somatic chromosome 3p deletions affecting both *VHL* and other chromosome 3p TSGs, such as *BAP1*, *PBRM1* and *SETD2* (37). In contrast, *ELOC*-mutated RCCs have a high frequency of chromosome 8 deletions, but chromosome 3p deletions are infrequent. While these patterns of chromosomal loss reflect the occurrence of ‘second hit’ deletion events in the two categories of RCC, it is interesting that there are not more similarities in the somatic mutation patterns outside of *VHL*/*ELOC*. These differences in tumour evolution may lead to differences in tumour growth patterns; e.g. *VHL*-related RCC may show gain of chromomere 8q, including amplification of MYC, which has been associated with a more aggressive tumour phenotype (37–39) and a more indolent course of *ELOC*-mutated RCC has been suggested previously (20). In addition, differences in the copy number profiles and pathological appearances of *VHL*- and *ELOC*-mutated RCCs could be utilized to differentiate between *ELOC*-associated VHL disease and classical *VHL*-related VHL disease.

Given the effect of inactivation of ELOC on the function of the VBC-CR, a VHL phenotype being associated with germline p.Tyr79Cys is perhaps not unexpected. However, at this stage, it is unclear whether germline *ELOC* mutations will solely mimic VHL disease or will be associated with other clinical phenotypes. The presence of parathyroid adenomas at a young age is not a known feature of VHL disease and this may be coincidental in our case. The haemangioblastoma and two RCCs from the proband showed typical features of those associated with germline *VHL* mutations. In addition, on pathology review, the presence of a leiomyomatous stroma and occasional branched tubular structures lined by cells with voluminous cytoplasm, features of RCC with somatic *ELOC* mutations, were noted focally in the RCC (Fig. 2A–C) (20,21). Currently, we would suggest that testing of *ELOC* should be performed in patients with suspected VHL disease but without an identifiable *VHL* mutation. The clinical course of *ELOC*-mutated RCC is variable (21); however, based on existing data, we would propose that individuals with a pathogenic germline variant should be managed as per VHL disease (40). While the emphasis of VHL management is primarily early diagnosis and treatment, the mechanistic similarities between *VHL*- and *ELOC* p.Tyr79Cys-associated tumours suggest that treatment with HIF-2α antagonists, such as bezultifan, may be a therapeutic option for *ELOC*-mutated tumours (41).

## Materials and Methods

### Patient ascertainment

All subjects gave written informed consent for genetic studies; the investigations were approved by the South Birmingham Research Ethics committee and were conducted in accordance with the Declaration of Helsinki. Participants from the 100,000 Genomes Project were consented as per the 100,000 Genomes Project protocol (29).

### Germline sequencing

DNA was extracted from peripheral blood samples of patients according to standard protocols. WES was performed in-house using Illumina DNA Prep with Enrichment (formerly named Nextera Flex for Enrichment) (42) on Illumina’s HiSeq 4000 platform with 150 bp paired end reads. Raw Illumina BCL files were demultiplexed and converted to FASTQ format using Illumina’s bcl2fastq version 2.19. All sample pairs were aligned to the hg38 version of the reference human genome using BWA-0.7.15 as previously described (43). The generated SAM file was compressed into a BAM file and sorted by genomic position using SAMtools version 1.3.1 (44). The sorted BAM files were subject to Base Quality Score Recalibration and Indel Realignment followed by variant calling using the Haplotype Caller algorithm as specified in the Genome-Analysis Toolkit (GATK) version 3.8 best practices (45–47). VCF files were filtered for a minimum depth of 20 reads and a Genotype Quality of 30 using VCFtools version 0.1.15 (48). VCF files were annotated with ANNOVAR (49).

Trio analysis in the proband and parents identified 126 exonic *de novo* variants in the proband. After filtering for rare exonic *de novo* variants (The Genome Aggregation Database maximum allele frequency ≤ 0.5%), 16 exonic *de novo* variants were further analyzed (Supplementary Material, Table S1).

Deep intronic and promoter *VHL* variants, described previously in VHL disease or erythrocytosis, were excluded from WGS data available for the proband (Supplementary Material, Table S2). WGS for the 100,000 Genomes Project participants was performed according to the 100,000 Genomes Project protocol (29). Whole-genome 150 bp paired-end TruSeq PCR-free libraries were sequenced on a single lane using Illumina (San Diego, USA) HiSeq X technology and were uniformly processed on the Illumina North Star Version 4 Whole Genome Sequencing Workflow (NSV4, version 2.6.53.23). Raw sequencing data were aligned to the NCBI GRCh38 assembly (with decoys) using iSAAC Aligner (version 03.16.02.19) and small germline variants were called using Starling (version 2.4.7). VCF files from WGS were annotated using VEP version 99 (50).

SNVs, CNVs and SVs in *AIP*, *CDC73*, *CDKN1B*, *MEN1* and *RET* were also excluded from WES data available for the proband. SVs and CNVs were annotated with BEDTools (51). SVs were called with Delly v0.8.1 (52) and CNVs were called with GATK4 version 4.1.4.0 best practices (45,53).

Targeted Sanger sequencing (*n* = 25) and exome sequencing (*n* = 66), on DNA extracted from blood in cohorts of patients previously examined for germline mutations in *VHL* without a mutation, were performed to determine the likely frequency of germline variants in *ELOC.* Previous clinical testing using Sanger sequencing analysis, MLPA and methylation analysis of *VHL* had not identified a pathogenic SNV or CNV in any of the samples.

### Tumour studies

Targeted tumour sequencing was performed on the DNA pair extracted from the proband’s macro-dissected formalin-fixed paraffin-embedded right kidney tumour specimen and DNA extracted from blood (germline). Library preparation was performed using Illumina DNA Prep with Enrichment (42)on Illumina’s HiSeq 4000 platform. Paired WES for tumour/germline DNA was analyzed for CNVs and SNVs/indels. SNV and SV analyses were performed as described earlier. aCGH was performed on the paired tumour/germline DNA samples using Illumina’s 750K SNP genotyping array (54).

### Sanger sequencing of germline samples

Sanger sequencing was performed using standard techniques, as per the Eurofins protocol (55). The following primer pairs were used:

*ELOC* exon 1: 5′-ccacccctagatggcttgaa-3′, 3′-tgcaaacgacgctttatagtc-5′,

*ELOC* exon 2: 5′-gtgggtggatcatgaggtca-3′, 3′-cagtttcttctgcaaaagctgt-5′,

*ELOC* exon 3: 5′-tttgagaccagcctgaccaa-3′, 3′-agctgtacctagtaaccttcca-5′,

*ELOC* exon 4: 5′-aaaattagccggtcgtggtg-3′, 3′-cttctgcaaaagctgtacctagt-5′.

The following conditions were used: (i) 95°C for 30 s, (ii) 60°C for 30 s, (iii) 72°C for 45 s, (iv) repeat (i)–(iii) for 30 times and (v) incubate at 72°C for 10 min.

## Supplementary Material

ELOC_suppl_informationt_HMG_revised_ddac066
