# Supplementary material for: Elongin C (ELOC/TCEB1)-associated von Hippel–Lindau disease
Source: Hum Mol Genet. 2022 Mar 21;31(16):2728–37. doi: 10.1093/hmg/ddac066 (PMC9402235; doi:10.1093/hmg/ddac066)
Supplement: ELOC_suppl_informationt_HMG_revised_ddac066 [file eloc_suppl_informationt_hmg_revised_ddac066.docx]

**Supplementary Material**

**Elongin C (*ELOC/TCEB1*) associated von Hippel-Lindau disease**

Avgi Andreou,^1^ Bryndis Yngvadottir,^1^ Laia Bassaganyas,^1^ Graeme Clarke,^1,2^ Ezequiel Martin,^1,2^ James Whitworth,^1^ Alex J Cornish,^3^ Genomics England Research Consortium, Richard S Houlston,^3^ Philip Rich,^4^ Catherine Egan,^5^ Shirley V Hodgson,^6^ Anne Y Warren,^7^ Katie Snape,^8^ Eamonn R Maher^1,*^

**^*^Corresponding author**

**Author affiliations:**

1. Department of Medical Genetics, University of Cambridge, Cambridge Biomedical Campus, Cambridge, CB2 0QQ, UK
2. Stratified Medicine Core Laboratory NGS Hub, Cambridge Biomedical Campus, Cambridge, CB2 0QQ, UK
3. Division of Genetics and Epidemiology, The Institute of Cancer Research, Sutton, Surrey, SM2 5NG, UK.
4. Department of Neuroradiology, St George's University Hospitals NHS Foundation Trust, London, SW17 0QT, UK
5. Moorfields Eye Hospital NHS Foundation Trust, London, EC1V 2PD, UK
6. Department of Medical Genetics, St. George's University of London, London, SW17 0QT, UK
7. Department of Histopathology, Cambridge University NHS Foundation Trust, Cambridge CB2 OQQ, UK
8. Department of Clinical Genetics, St George's University Hospitals NHS Foundation Trust and St George's University of London, London, SW17 0QT, UK

**Corresponding author:**

Professor Eamonn R Maher, Department of Medical Genetics, University of Cambridge, Box 238, Cambridge Biomedical Campus, Cambridge, CB2 0QQ, UK, +44 01223 746715, [erm1000@medschl.cam.ac.uk](mailto:erm1000@medschl.cam.ac.uk).

**Supplementary Figures**

## **Supplementary Figure 1:** Comparison of WES alignments of DNA extracted from proband´s kidney tumour specimen and blood (germline).

The Binary Alignment Map (BAM) files were viewed with the Integrative Genomics Viewer (IGV) (1).

#
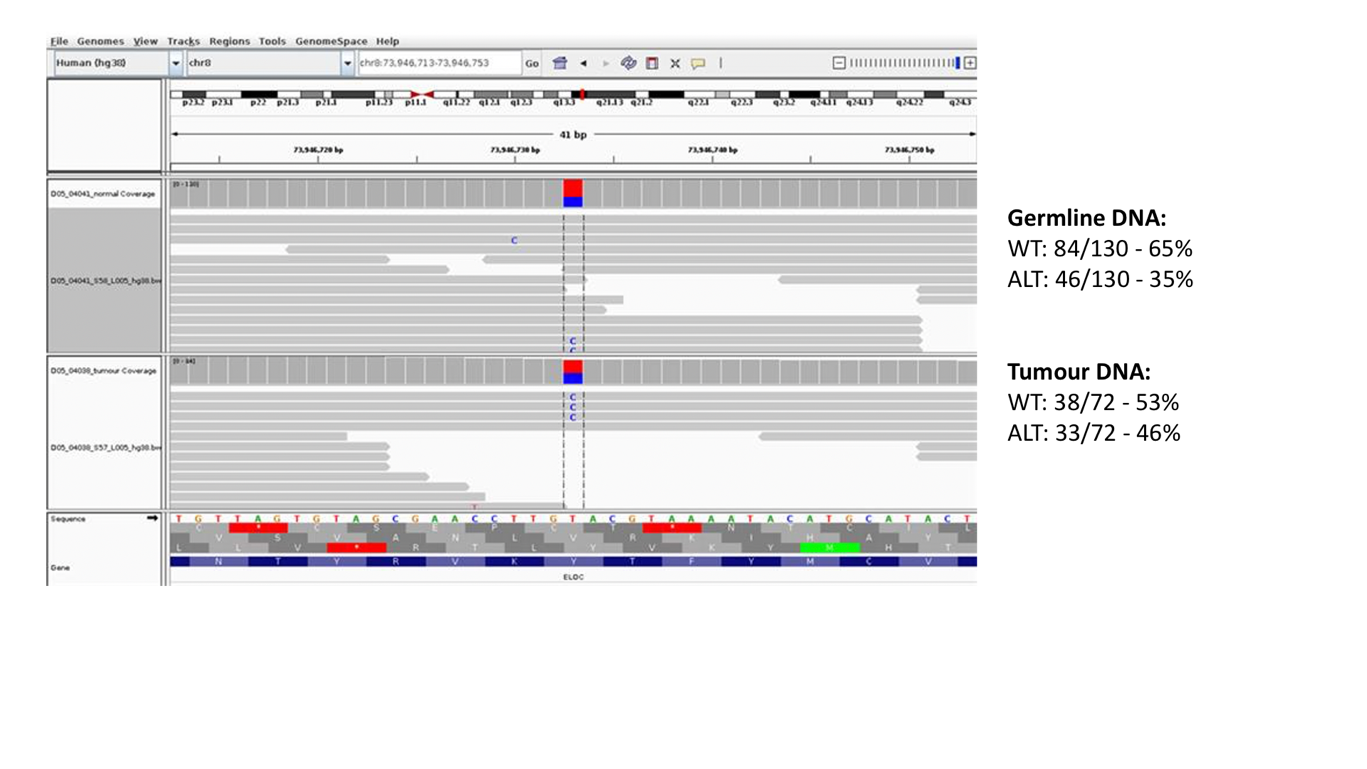


WT= wild type allele, ALT= alternative allele

# **Supplementary Tables**

## **Supplementary Table 1:** Rare *de novo* variants in proband.

| Gene | Consequence | HGVSc^[[1]](#footnote-1)^ | HGVSp^[[2]](#footnote-2)^ | dbSNP^[[3]](#footnote-3)^ | gnomAD_ALL^[[4]](#footnote-4)^ |
| --- | --- | --- | --- | --- | --- |
| *CRIPAK* | frameshift insertion | c.51_52insTGCCCATGTGGAGTGCCCGCCTGCTCACACA | p.T17fs | rs750778284 | 0 |
| *FGFRL1* | frameshift deletion | c.1435_1436del | p.H479fs | rs145808953 | 1.10x10^-3^ |
| *TMEM185B* | frameshift deletion | c.1023delT | p.P341fs |  | 0 |
| *HEG1* | nonsynonymous SNV | c.1232G>A | p.R411H | rs538286740 | 6.45x10^-5^ |
| *PABPC1* | nonsynonymous SNV | c.541G>A | p.A181T | rs201575415 | 9.76x10^-5^ |
| *PABPC1* | nonsynonymous SNV | c.619C>T | p.L207F | rs200538577 | 9.76x10^-5^ |
| *HLA-DRB5* | nonsynonymous SNV | c.197A>C | p.D66A | rs200042906 | 2.70x10^-3^ |
| *PABPC1* | nonsynonymous SNV | c.617G>A | p.R206H | rs201157005 | 6.51x10^-5^ |
| *PDE4DIP* | nonsynonymous SNV | c.4186C>T | p.R1396W | rs2798901 | 0 |
| *ATAD3B* | nonsynonymous SNV | c.1907G>C | p.G636A | rs553799027 | 9.73x10^-5^ |
| *CELF3* | nonframeshift deletion | c.1098_1100del | p.366_367del | rs777856157 | 6.52x10^-5^ |
| *HRNR* | nonsynonymous SNV | c.5161G>A | p.G1721S |  | 0 |
| *OTOP1* | nonsynonymous SNV | c.310C>A | p.L104M | rs200554408 | 0 |
| *SSPO* | stopgain | c.11582dupA | p.Y3861_C3862delinsX |  | 0 |
| *PAXIP1* | nonframeshift deletion | c.1642_1644del | p.548_548del | rs141168451 | 6.54x10^-5^ |
| *ELOC* | nonsynonymous SNV | c.236A>G | p.Y79C |  | 0 |

**Supplementary Table 2:** List of deep intronic and promoter variants in *VHL* excluded from the proband.

| Variants | dbSNP | Type of variant | Consequence | Phenotype | Publication |
| --- | --- | --- | --- | --- | --- |
| NM_000551.4(VHL):c.340+770T>C | rs1346312258 | cryptic exon (E1') variant in intron 1 | splicing dysregulation | erythrocytosis | Lenglet et. al. 2018 (2) |
| NM_000551.4(VHL):c.340+694_340+711dup | rs1575923363 | cryptic exon (E1') variant in intron 1 | splicing dysregulation | erythrocytosis | Lenglet et. al. 2018 (2) |
| NM_000551.4(VHL):c.340+574A>T | rs98274567 | cryptic exon (E1') variant in intron 1 | splicing dysregulation | erythrocytosis | Lenglet et. al. 2018 (2) |
| NM_000551.4(VHL):c.340+816A>C | rs1031288121 | cryptic exon (E1') variant in intron 1 | splicing dysregulation | erythrocytosis | Lenglet et. al. 2018 (2) |
| NM_000551.4(VHL):c.340+617C>G | rs1575923261 | cryptic exon (E1') variant in intron 1 | splicing dysregulation | VHL syndrome | Lenglet et. al. 2018 (2) |
| NM_000551.4(VHL):c.340+648T>C | rs73024533 | cryptic exon (E1') variant in intron 1 | splicing dysregulation | VHL syndrome | Lenglet et. al. 2018 (2) |
| NM_000551.4(VHL):c.340+665G>C | rs1696160266 | nearby variant to c.340+648T>C | splicing dysregulation | functional work evidence only | Lenglet et. al. 2018 (2) |
| NM_000551.4(VHL):c.429C>T (p.Asp143=) | rs773556807 | synonymous variants in exon 2 that induce exon skipping | exon skipping | erythrocytosis | Lenglet et. al. 2018 (2) |
| NM_000551.4(VHL):c.414A>G (p.Pro138=) | rs869025648 | synonymous variants in exon 2 that induce exon skipping | exon skipping | VHL syndrome, phaeochromocytoma | Lenglet et. al. 2018, Flores et. al. 2019 (2, 3) |
| NM_000551.4(VHL):c.413C>T (p.Pro138Leu) | rs780178275 | nearby variant to c.414A>G | splicing dysregulation | functional work evidence only | Lenglet et. al. 2018 (2) |
| NM_000551.3(VHL):c.-75_-55del | rs727503744 | promoter/5'UTR variant | promoter/5'UTR variant | VHL syndrome | Zatyka et. al. 2002 (4) |
| NM_000551.4(VHL):c.-54_-35dup | rs730882036 | promoter/5'UTR variant | does not appear to affect the start codon or the Kozak translational consensus sequence. | VHL syndrome | Landrum et. al. 2016 (5); Accession: SCV000211830.2 |
| NM_000551.3(VHL):c.-77_-32del | rs1553619239 | 5'UTR variant including the promoter | Reduced *VHL* expression | VHL syndrome | Albanyan et. al. 2019 (6) |
| NM_000551.4(VHL):c.-61_-51dup | rs727503743 | upstream 5'UTR variant | Mild reduction in *VHL* expression | VHL-like disease | Albanyan et. al. 2019 (6) |

## **Supplementary Table 3:** Phenotypes of individuals with multiple VHL-related tumours or a single VHL-related tumour plus a family history of a VHL-related tumour tested for germline ELOC variants.

| **ID** | | **Class** | | **Sex** | | **Age** | | **Clinical details** | | **Testing** | |
| --- | --- | --- | --- | --- | --- | --- | --- | --- | --- | --- | --- |
| Fam_1 | | VHLSp | | N/A | | N/A | | Familial RCC | | WES | |
| Fam_2 | | VHLSp | | N/A | | 71 | | Familial RCC | | WES | |
| Fam_3 | | VHLSp | | N/A | | N/A | | Familial RCC | | WES | |
| Fam_4 | | VHLSp | | F | | 60 | | Familial RCC | | WES | |
| Fam_5 | | VHLSp | | M | | 62 | | Familial RCC | | WES | |
| Fam_6 | | VHLSp | | N/A | | 55 | | Familial RCC | | WES | |
| Fam_7 | | VHLSp | | N/A | | 56 | | Familial RCC | | WES | |
| Fam_8 | | VHLSp | | N/A | | 57 | | Familial RCC | | WES | |
| Fam_9 | | VHLSp | | N/A | | 64 | | Familial RCC | | WES | |
| Fam_10 | | VHLSp | | N/A | | 56 | | Familial RCC | | WES | |
| Fam_11 | | VHLSp | | F | | 60 | | Bilateral/multifocal RCC | | WES | |
| Fam_12 | | VHLSp | | M | | 52 | | Familial RCC | | WES | |
| Fam_13 | | VHLSp | | F | | 52 | | Familial RCC | | WES | |
| Fam_15 | | VHLSp | | M | | 58 | | Bilateral/multifocal RCC | | WES | |
| Fam_16 | | VHLSp | | M | | 48 | | Bilateral/multifocal RCC | | WES | |
| Fam_17 | | VHLSp | | M | | 41 | | Familial RCC | | WES | |
| Fam_18 | | VHLSp | | M | | 49 | | Bilateral/multifocal RCC | | WES | |
| Fam_19 | | VHLSp | | F | | 47 | | Familial RCC | | WES | |
| Fam_20 | | VHLSp | | N/A | | 51 | | Bilateral/multifocal RCC | | WES | |
| Fam_21 | | VHLSp | | M | | 49 | | Familial RCC | | WES | |
| Fam_22 | | VHLSp | | F | | 32 | | Bilateral/multifocal RCC | | WES | |
| Fam_23 | | VHLSp | | M | | 49 | | Familial RCC | | WES | |
| Fam_24 | | VHLSp | | M | | 40 | | Familial RCC | | WES | |
| Fam_25 | | VHLSp | | M | | 38 | | Bilateral/multifocal RCC | | WES | |
| Fam_26 | | VHLSp | | F | | 40 | | Bilateral/multifocal RCC | | WES | |
| Fam_27 | | VHLSp | | M | | 41 | | Familial RCC | | WES | |
| Fam_28 | | VHLSp | | M | | 31 | | Bilateral/multifocal RCC | | WES | |
| Fam_29 | | VHLSp | | N/A | | 30 | | Familial RCC | | WES | |
| Fam_30 | | VHLSp | | M | | 45 | | Bilateral/multifocal RCC | | WES | |
| Fam_31 | | VHLSp | | M | | 53 | | Bilateral/multifocal RCC | | WES | |
| Fam_32 | | VHLSp | | M | | 38 | | Familial RCC | | WES | |
| Fam_33 | | VHLSp | | M | | 48 | | Bilateral/multifocal RCC | | WES | |
| Fam_34 | | VHLSp | | M | | 42 | | Familial RCC | | WES | |
| Fam_35 | | VHLSp | | M | | 34 | | Familial RCC | | WES | |
| Fam_36 | | VHLSp | | M | | 54 | | Familial RCC | | WES | |
| Fam_37 | | VHLSp | | M | | 48 | | Bilateral/multifocal RCC | | WES | |
| Fam_38 | | VHLSp | | N/A | | 45 | | Familial RCC | | WES | |
| Fam_39 | | VHLSp | | F | | 61 | | Bilateral/multifocal RCC | | WES | |
| Fam_40 | | VHLSp | | M | | 50 | | Bilateral/multifocal RCC | | WES | |
| Fam_41 | | VHLSp | | F | | 50 | | Familial RCC | | WES | |
| Fam_42 | | VHLSp | | M | | 39 | | Familial RCC | | WES | |
| Fam_43 | | VHLSp | | M | | 30 | | Familial RCC | | WES | |
| Fam_44 | VHLSp | | N/A | | 74 | | Familial RCC | | WES | |  |
| Fam_45 | VHLSp | | M | | 48 | | Familial RCC | | WES | |  |
| Fam_46 | VHLSp | | M | | 61 | | Familial RCC | | WES | |  |
| Fam_47 | VHLSp | | F | | 47 | | Bilateral/multifocal RCC | | WES | |  |
| Fam_48 | VHLSp | | M | | 72 | | Bilateral/multifocal RCC | | WES | |  |
| Fam_49 | VHLSp | | M | | 57 | | Familial RCC | | WES | |  |
| Fam_50 | VHLSp | | F | | 40 | | Bilateral/multifocal RCC | | WES | |  |
| Fam_51 | VHLSp | | M | | 69 | | Familial RCC | | WES | |  |
| Fam_52 | VHLSp | | M | | 42 | | Bilateral/multifocal RCC | | WES | |  |
| Fam_53 | VHLSp | | M | | 46 | | Bilateral/multifocal RCC | | WES | |  |
| Fam_54 | VHLSp | | M | | 46 | | Bilateral/multifocal RCC | | WES | |  |
| Fam_55 | VHLSp | | F | | 11 | | Familial RCC | | WES | |  |
| Fam_56 | VHLSp | | M | | 37 | | Bilateral/multifocal RCC | | WES | |  |
| Fam_57 | VHLSp | | M | | 63 | | PHEO, RCC | | WES | |  |
| Fam_58 | VHLSp | | F | | 46 | | Familial PHEO | | WES | |  |
| Fam_59 | VHLSp | | F | | 52 | | Familial PHEO | | WES | |  |
| Fam_60 | VHLSp | | F | | 41 | | Bilateral/multifocal RCC | | WES | |  |
| Fam_61 | VHLSp | | F | | 45 | | Bilateral/multifocal RCC | | WES | |  |
| Fam_62 | VHLSp | | N/A | | N/A | | Familial RCC | | WES | |  |
| Fam_63 | VHLSp | | N/A | | 62 | | Bilateral/multifocal RCC | | WES | |  |
| Fam_64 | VHLSp | | N/A | | 55 | | Bilateral/multifocal RCC | | WES | |  |
| Fam_65 | VHLSp | | N/A | | 47 | | Familial RCC | | WES | |  |
| Fam_66 | VHLSp | | N/A | | 59 | | Bilateral/multifocal RCC | | WES | |  |
| Fam_67 | VHLSp | | M | | N/A | | HB, PHEO | | Targeted | |  |
| Fam_68 | VHLSp | | F | | N/A | | RA, HB | | Targeted | |  |
| Fam_69 | VHLSp | | M | | N/A | | HB, RCC | | Targeted | |  |
| Fam_70 | VHLSp | | N/A | | N/A | | RA,HB | | Targeted | |  |
| Fam_71 | VHLSp | | N/A | | N/A | | Clinical VHL diagnosis | | Targeted | |  |
| Fam_72 | VHLSp | | N/A | | N/A | | Clinical VHL diagnosis | | Targeted | |  |
| Fam_73 | VHLSp | | N/A | | N/A | | Clinical VHL diagnosis | | Targeted | |  |
| Fam_74 | VHLSp | | N/A | | N/A | | Clinical VHL diagnosis | | Targeted | |  |
| Fam_75 | VHLSp | | N/A | | N/A | | Clinical VHL diagnosis | | Targeted | |  |
| Fam_76 | VHLSp | | N/A | | N/A | | Clinical VHL diagnosis | | Targeted | |  |
| Fam_77 | VHLSp | | N/A | | N/A | | Clinical VHL diagnosis | | Targeted | |  |
| Fam_78 | VHLSp | | N/A | | N/A | | Clinical VHL diagnosis | | Targeted | |  |
| Fam_79 | VHLSp | | N/A | | N/A | | Clinical VHL diagnosis | | Targeted | |  |
| Fam_80 | VHLSp | | N/A | | N/A | | Clinical VHL diagnosis | | Targeted | |  |
| Fam_81 | VHLSp | | N/A | | N/A | | Clinical VHL diagnosis | | Targeted | |  |
| Fam_82 | VHLSp | | N/A | | N/A | | Clinical VHL diagnosis | | Targeted | |  |
| Fam_83 | VHLSp | | N/A | | N/A | | Clinical VHL diagnosis | | Targeted | |  |
| Fam_84 | VHLSp | | N/A | | N/A | | Clinical VHL diagnosis | | Targeted | |  |
| Fam_85 | VHLSp | | N/A | | N/A | | Clinical VHL diagnosis | | Targeted | |  |
| Fam_86 | VHLSp | | N/A | | N/A | | Clinical VHL diagnosis | | Targeted | |  |
| Fam_87 | VHLSp | | N/A | | N/A | | Clinical VHL diagnosis | | Targeted | |  |
| Fam_88 | VHLSp | | N/A | | N/A | | Clinical VHL diagnosis | | Targeted | |  |
| Fam_89 | VHLSp | | N/A | | N/A | | Clinical VHL diagnosis | | Targeted | |  |
| Fam_90 | VHLSp | | N/A | | N/A | | Clinical VHL diagnosis | | Targeted | |  |
| Fam_91 | VHLSp | | N/A | | N/A | | Clinical VHL diagnosis | | Targeted | |  |

VHLSp=VHL spectrum phenotype, PHEO= pheochromocytoma, RCC= renal cell carcinoma, HB=haemangioblastoma, RA=retinal angioma

## **Supplementary Table 4:** RCC somatic *ELOC* variants reported in different studies.

| Cohort | Variant | Variant type | Count | Publication |
| --- | --- | --- | --- | --- |
| TCGA^[[5]](#footnote-5)^ | Y79C | missense | 3 | Ricketts et. el. 2018 (7) |
| TCGA | Y79N | missense | 1 | Ricketts et. el. 2018 (7) |
| TCGA | Y79S | missense | 1 | Ricketts et. el. 2018 (7) |
| TCGA | Y79F | missense | 1 | Ricketts et. el. 2018 (7) |
| TCGA | Y79* | nonsense | 1 | Ricketts et. el. 2018 (7) |
| TCGA | Y79* | nonsense | 1 | Ricketts et. el. 2018 (7) |
| TCGA | C112Vfs*3 | frameshift deletion | 1 | Ricketts et. el. 2018 (7) |
| Sato *et al* 2013 | Y79C | missense | 6 | Sato et. al. 2013 (8) |
| Sato *et al* 2013 | Y79S | missense | 1 | Sato et. al. 2013 (8) |
| Sato *et al* 2013 | A100P | missense | 1 | Sato et. al. 2013 (8) |
| TRACERx Renal^[[6]](#footnote-6)^ | K114R | missense | 1 | Hakimi et. al. 2015 (9) |
| TRACERx Renal | I95N | missense | 1 | Mitchell et. al. 2018 (10) |
| MSKCC^[[7]](#footnote-7)^ | Y79C/S/L/N | missense | 5 | DiNatale et. al. 2021 (11) |

## **Supplementary Table 5:** RCC somatic *ELOC* variants from the 100,000 Genomes Project.

| Case no | Gene | HGVSc^[[8]](#footnote-8)^ | HGVSp^[[9]](#footnote-9)^ | CHR POS (GRCh38) | REF | ALT | Consequence |
| --- | --- | --- | --- | --- | --- | --- | --- |
| Case 1 | ELOC | ENST00000284811.12:  c.236A>G | ENSP00000284811.8:  p.Tyr79Cys | 8:73946733 | T | C | missense |
| Case 2 | ELOC | ENST00000284811.12:  c.236A>G | ENSP00000284811.8:  p.Tyr79Cys | 8:73946733 | T | C | missense |
| Case 3 | ELOC | ENST00000284811.12:  c.236A>G | ENSP00000284811.8: p.Tyr79Cys | 8:73946733 | T | C | missense |
| Case 4 | ELOC | ENST00000284811.12:  c.236A>G | ENSP00000284811.8: p.Tyr79Cys | 8:73946733 | T | C | missense |
| Case 5 | ELOC | ENST00000284811.12: c.274G>A | ENSP00000284811.8: p.Glu92Lys | 8:73946695 | C | T | missense |
| Case 6 | ELOC | ENST00000284811.12:  c.74A>T | ENSP00000284811.8: p.Asp25Val | 8:73955985 | T | A | missense |
| Case 7 | ELOC | ENST00000284811.12:  c.311T>A | ENSP00000284811.8: p.Leu104Gln | 8:73946658 | A | T | missense |
| Case 8 | ELOC | ENST00000284811.12:  c.261_272del | ENSP00000284811.8:  p.Thr88_Pro91del | 8:73946696 | AGGAATCTCGGTG | A | Inframe deletion |

# **Supplementary References**

1 Robinson, J.T., Thorvaldsdottir, H., Winckler, W., Guttman, M., Lander, E.S., Getz, G. and Mesirov, J.P. (2011) Integrative genomics viewer. *Nat. Biotechnol.*, **29**, 24-26.

2 Lenglet, M., Robriquet, F., Schwarz, K., Camps, C., Couturier, A., Hoogewijs, D., Buffet, A., Knight, S.J.L., Gad, S., Couve, S. *et al.* (2018) Identification of a new VHL exon and complex splicing alterations in familial erythrocytosis or von Hippel-Lindau disease. *Blood*, **132**, 469-483.

3 Flores, S.K., Cheng, Z., Jasper, A.M., Natori, K., Okamoto, T., Tanabe, A., Gotoh, K., Shibata, H., Sakurai, A., Nakai, T. *et al.* (2019) A synonymous VHL variant in exon 2 confers susceptibility to familial pheochromocytoma and von Hippel-Lindau disease. *J. Clin. Endocrinol. Metab.*, **104**, 3826-3834.

4 Zatyka, M., Morrissey, C., Kuzmin, I., Lerman, M.I., Latif, F., Richards, F.M. and Maher, E.R. (2002) Genetic and functional analysis of the von Hippel-Lindau (VHL) tumour suppressor gene promoter. *J. Med. Genet.*, **39**, 463-472.

5 Landrum, M.J., Lee, J.M., Benson, M., Brown, G.R., Chao, C., Chitipiralla, S., Gu, B., Hart, J., Hoffman, D., Jang, W. *et al.* (2018) ClinVar: improving access to variant interpretations and supporting evidence. *Nucleic Acids. Res.*, **46**, D1062-D1067.

6 Albanyan, S., Giles, R.H., Gimeno, E.M., Silver, J., Murphy, J., Faghfoury, H., Morel, C.F., Machado, J. and Kim, R.H. (2019) Characterization of VHL promoter variants in patients suspected of Von Hippel-Lindau disease. *Eur. J. Med. Genet.*, **62**, 177-181.

7 Ricketts, C.J., De Cubas, A.A., Fan, H., Smith, C.C., Lang, M., Reznik, E., Bowlby, R., Gibb, E.A., Akbani, R., Beroukhim, R. *et al.* (2018) The Cancer Genome Atlas Comprehensive Molecular Characterization of Renal Cell Carcinoma. *Cell Rep.*, **23**, 313-326 e315.

8 Sato, Y., Yoshizato, T., Shiraishi, Y., Maekawa, S., Okuno, Y., Kamura, T., Shimamura, T., Sato-Otsubo, A., Nagae, G., Suzuki, H. *et al.* (2013) Integrated molecular analysis of clear-cell renal cell carcinoma. *Nat. Genet.*, **45**, 860-867.

9 Hakimi, A.A., Tickoo, S.K., Jacobsen, A., Sarungbam, J., Sfakianos, J.P., Sato, Y., Morikawa, T., Kume, H., Fukayama, M., Homma, Y. *et al.* (2015) TCEB1-mutated renal cell carcinoma: a distinct genomic and morphological subtype. *Mod. Pathol.*, **28**, 845-853.

10 Mitchell, T.J., Turajlic, S., Rowan, A., Nicol, D., Farmery, J.H.R., O'Brien, T., Martincorena, I., Tarpey, P., Angelopoulos, N., Yates, L.R. *et al.* (2018) Timing the Landmark Events in the Evolution of Clear Cell Renal Cell Cancer: TRACERx Renal. *Cell*, **173**, 611-623 e617.

11 DiNatale, R.G., Gorelick, A.N., Makarov, V., Blum, K.A., Silagy, A.W., Freeman, B., Chowell, D., Marcon, J., Mano, R., Sanchez, A. *et al.* (2021) Putative Drivers of Aggressiveness in TCEB1-mutant Renal Cell Carcinoma: An Emerging Entity with Variable Clinical Course. *Eur. Urol. Focus*, **7**, 381-389.

1. Human Genome Variation Society coding sequence name [↑](#footnote-ref-1)
2. Human Genome Variation Society protein sequence name [↑](#footnote-ref-2)
3. Single Nucleotide Polymorphism Database number [↑](#footnote-ref-3)
4. maximum allele frequency from all ethnicities in gnomAD database [↑](#footnote-ref-4)
5. TRACERx Renal= TRAcking Cancer Evolution through therapy (Rx) Renal cohort [↑](#footnote-ref-5)
6. MSKCC= Memorial Sloan Kettering Cancer Centre [↑](#footnote-ref-6)
7. PMID= Pubmed ID [↑](#footnote-ref-7)
8. Human Genome Variation Society coding sequence name [↑](#footnote-ref-8)
9. Human Genome Variation Society protein sequence name [↑](#footnote-ref-9)
